# Angiogenesis-Related Cytokines, RANKL, and Osteoprotegerin in Multiple Myeloma Patients in relation to Clinical Features and Response to Treatment

**DOI:** 10.1155/2011/867576

**Published:** 2011-09-18

**Authors:** K. Sfiridaki, C. A. Pappa, G. Tsirakis, P. Kanellou, M. Kaparou, M. Stratinaki, G. Sakellaris, G. Kontakis, M. G. Alexandrakis

**Affiliations:** ^1^Blood Bank, Venizelion General Hospital of Heraklion, 71409 Crete, Greece; ^2^Department of Haematology, University Hospital of Heraklion, 70013 Crete, Greece; ^3^Department of Surgery, University Hospital of Heraklion, 70013 Crete, Greece

## Abstract

An essential cytokine system for the osteoclast biology in multiple myeloma (MM) consists of the receptor of activator of NF-*κ*B ligand (RANKL), its receptor (RANK), and the soluble decoy receptor, osteoprotegerin (OPG). Myeloma cells cause imbalance in OPG/RANKL interactions. We measured serum levels of OPG, soluble (s) RANKL, sRANKL/OPG ratio, markers of disease activity [LDH, CRP, interleukin-6 (IL-6), *β*2-microglobulin (B2M)], and angiogenic factors [hepatocyte growth factor (HGF), vascular endothelial growth factor (VEGF)], in 54 newly diagnosed MM patients and in 25 of them in plateau phase. All the above values were higher in MM patients compared to controls and decreased in plateau phase. sRANKL and RANKL/OPG were higher with advancing disease stage and skeletal grade. Significant correlations were found among RANKL and RANKL/OPG with HGF, LDH, VEGF, IL-6, and B2M. In conclusion, RANKL and OPG play significant roles in MM pathophysiology, as regulators of bone turnover and mediators of angiogenesis.

## 1. Introduction

Multiple myeloma is a malignant plasma cell proliferation localized in the bone marrow with debilitating skeletal involvement. Much of its morbidity is accounted to bone pain and pathologic fractures due to the imbalance between bone formation and breakdown in favor of the latter [1–4]. 

These complications are due to an excessive osteoclast activity, which are stimulated by osteoclast-activating factors, including Tumor Necrosis Factor (TNF), Interleukin-1 (IL-1), Interleukin-6 (IL-6), the chemokines Macrophage Inflammatory Protein-1a (MIP-1a), Macrophage Inflammatory Protein-1*β* (MIP-1*β*), and Stroma cell-Derived Factor-1a (SDF-1a) [2, 5, 6]. 

However, an essential cytokine system for the osteoclast biology has been identified and consists of the receptor of activator of NF-*κ*B ligand (RANKL), its receptor RANK, and the soluble decoy receptor, osteoprotegerin (OPG) [7–9]. The RANKL has been identified and associated with the enhancement in osteoblast-induced bone resorption, while OPG blocks RANKL and inhibits bone resorption [8, 10]. Myeloma plasma cells have been found to induce an imbalance in the OPG/RANKL interactions, increasing RANKL expression and decreasing OPG availability in the bone microenvironment, inducing an enhancement of the osteoclastic activation and increased bone resorption [8, 11].

RANKL stimulates osteoclast differentiation and activity and also inhibits osteoclast apoptosis. RANKL is mainly produced by osteoblastic lineage cells, immune cells, and some malignant cells [12]. 

Osteoprotegerin is a Tumor Necrosis Factor Receptor-2 (TNFR-2) and CD40 homologous protein, which lacks a transmembrane domain and is secreted into the extracellular space as a homodimeric glycoprotein [13, 14]. OPG was found in serum of peripheral blood in various forms, in complexes with soluble RANKL and a free non-bound portion. The heterogeneity of these circulating forms may account for the discrepancies for absolute values reported in the literature [11]. Based on the heterogeneity of circulating forms and other technical aspects, the protein used as standard and the suitable calibrator diluent could account for discrepant results that have been obtained by different groups in the literature [11, 15]. 

Bone marrow angiogenesis is an important additional process contributing to disease progress [16, 17]. Various angiogenic cytokines, such as vascular endothelial growth factor (VEGF) and hepatocyte growth factor (HGF) increase myeloma cell proliferation and accelerate bone resorption [16, 18]. 

The purpose of this study was to evaluate serum levels of OPG, soluble RANKL (sRANKL), the ratio sRANKL/OPG, VEGF, HGF, and known factors of disease activity at diagnosis and in plateau phase and to estimate the effect of treatment on their circulating levels in patients with MM.

## 2. Patients and Methods

### 2.1. Patients

Fifty-four MM patients (28 males, 26 females) were included in this study. The median age was 64 years (range 51–87 years). Patients were diagnosed and categorized according to the Durie-Salmon classification system [19]. Sixteen patients were classified as stage I, 19 as stage II, and 19 as stage III. The types of monoclonal protein were 21 IgG*κ*, 8 IgG*λ*, 7 IgA*κ*, 11 IgA*λ*, and light chain disease for 7 patients. Bone involvement was graded according to standard X-ray evaluation into two scores: low score in 26 patients overall, including patients with no lesions, one bone involved, or diffuse osteoporosis; and high score in 28 patients overall, including patients with lesions in more than one bone or presence of bone fracture. None of the patients had received any treatment with corticosteroids or biphosphonates before entering the study. Eighteen patients had normal radiographic findings of the skeleton, the rest displayed osteolytic lesions. Twenty-five of the patients achieved complete remission or very good partial remission after treatment with PAD (bortezomib, doxorubicin, dexamethasone) regimen followed by autologous stem cell transplantation and considered to be in plateau phase. Twenty age- and sex-matched healthy subjects (12 male and 8 female, mean age 63.6 ± 10.8 years) were recruited as controls among blood donors (up to 60 years of age) and people following a physical training program (over 60 years of age). Informed consent for the study was obtained from all subjects. The study was conducted with the understanding and the consent of the human subject. The responsible Ethical Committee has approved the experiments. 

### 2.2. Methods

Serum samples from patients were collected before starting treatment at diagnosis and during stable disease. Sera collected from patients and controls were aliquoted into separate vials and stored at −7°C. All assays were performed at the end of the study, in order to avoid interassay variability. 

The detection of VEGF, HGF, IL-6, sRANKL, and OPG in the serum was performed by a solid-phase sandwich ELISA, using monoclonal antibodies against VEGF, HGF, and IL-6 (Quantikine, R&D systems Inc. Mineapolis, MN, USA) and sRANKL and OPG antibodies (Bio Vendor-Laboratorni Medicina as Brno, Czech Republic). Measurements were performed according to the manufacturers' instructions. Briefly, antibodies specific for each growth factor (VEGF, HGF, IL-6, sRANKL, and OPG) were coated in microtitter wells. After the addition of controls and samples and a first incubation and washing, an enzyme-linked polyclonal antibody specific for each growth factor was added to each well. After an incubation time and the last washing step, the remaining conjugate is allowed to react with the substrate. During the next step, the reaction was stopped by addition of specific solution, and the absorbance of the resulting color is measured at 450 nm. Biochemical markers that were studied were C-reactive protein (CRP), *β*2-microglobulin (B2M), and serum lactate dehydrogenase (LDH). The above mentioned parameters were evaluated using conventional techniques. CRP was determined by nephelometry, B2M by radioimmunoassay, and serum LDH was determined using standard laboratory methods. Radiographic examination including skull, pelvis, long bones and cervical, thoracic, and lumbar spine was carried out in all patients as a routine staging procedure. Bone disease was graded according to Durie and Salmon criteria [19].

### 2.3. Statistical Analysis

Data are presented as means ± SD. The Mann-Whitney *U*-test was applied to evaluate any difference in serum cytokine levels in patients and healthy individuals. Nonparametric tests were also used (Mann-Whitney and Wilcoxon for independent and paired two samples statistics). The investigation of potential correlations among variables was carried out with the Spearman's rho correlation coefficient. *P*  values < 0.05 were considered to be statistically significant.

## 3. Results

**Figure 1:**
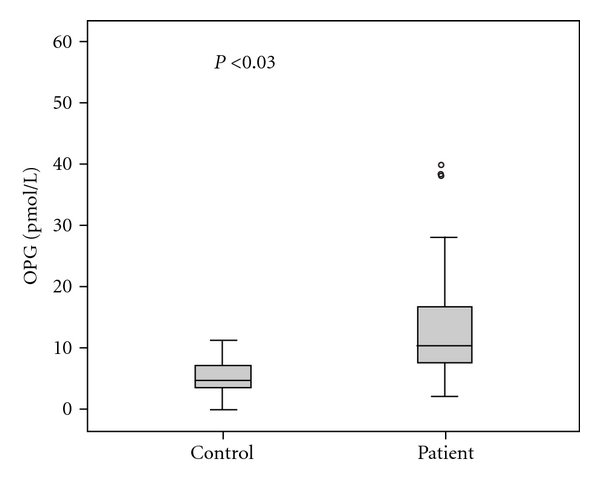
Serum OPG concentrations in MM patients and healthy controls.

**Figure 2:**
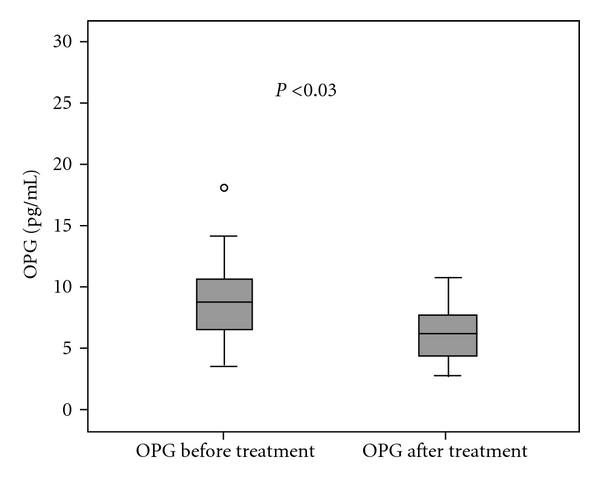
Serum OPG concentrations before and after treatment.

The laboratory findings studied in multiple myeloma patients and in healthy controls are shown in Table 1. Serum mean concentration of OPG was 12.5 ± 11.5 (0.7–60.2) pmol/L in multiple myeloma patients and 6.4 ± 5.3 (0.7–17.4) pmol/L in healthy subjects (*P* < 0.03) (by Mann-Whitney test) (Figure 1). The mean concentrations of OPG in stages I and II of the disease were higher than in stage III, without statistical significance. Our results also showed that OPG serum levels were significantly decreasing after treatment (11.5 ± 10.9 versus 13.3 ± 14.0 pmol/L, *P* < 0.03, Wilcoxon test) (Figure 2). Serum concentrations of sRANKL were also increased in patients compared to controls, whereas sRANKL/OPG ratio did not show significant difference. On the other hand, both serum levels of sRANKL and sRANKL/OPG ratio were increased with disease progression and decreased after effective treatment.

In healthy individuals, mean serum levels for HGF were 465.3 ± 163.4 (311.9–898.7) pg/mL, for VEGF 90.2 ± 14.4 (68.7–111.6) pg/mL and for IL-6 0.8 ± 0.5 (0.4–2.0) pg/mL. Pretreatment VEGF, HGF, and IL-6 serum levels were increased in MM patients in comparison with healthy individuals (*P* < 0.001, in all cases). Significant differences were found for HGF, VEGF, and IL-6 between the stages of the disease (Table 2). 

### 3.1. Relationship between Biochemical Markers, Cytokines, and Skeletal Involvement

**Figure 3:**
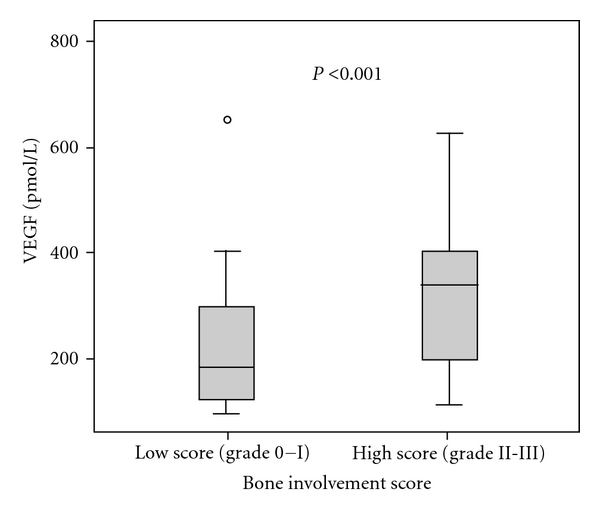
Serum VEGF levels according to bone involvement score.

There was a positive correlation between increasing bone lesion score and serum median concentrations of sRANKL (*P* < 0.001), sRANKL/OPG ratio (*P* < 0.001), VEGF (*P* < 0.001) (Figure 3), HGF (*P* < 0.004), IL-6 (*P* < 0.001), LDH (*P* < 0.05), and B2M (*P* < 0.04), while OPG levels according to bone disease were higher in low score (grade 0*-*1) in comparison to high score (grade 2*-*3) but without statistical significance (Table 3).

### 3.2. Correlation between Measured Parameters

Positive correlations were found between sRANKL with HGF, VEGF, LDH, VEGF, B2M, and IL-6, whereas more powerful correlations were found between sRANKL/OPG ratio with OPG and the above parameters (Table 4). Serum HGF correlated positively with LDH (*r* = 0.38, *P* < 0.002), VEGF (*r* = 0.59, *P* < 0.0001), B2M (*r* = 49, *P* < 0.0001), CRP (*r* = 420, *P* < 0.001), and IL-6 (*r* = 61, *P* < 0.0001). 

No correlation between the angiogenic cytokines VEGF, HGF and IL-6, and the serum OPG levels was found. Similarly, no correlation between the parameters of disease activity CRP, B2M, and LDH serum levels with OPG levels was found. 

Finally, statistically significant differences were observed in the serum levels, before and after treatment, of OPG, HGF, LDH, VEGF, and IL-6 (Table 5).

## 4. Discussion

Angiogenesis is a crucial factor for the growth and progression of multiple myeloma [16, 20, 21]. Various angiogenic cytokines have been characterized as potent mitogens with angiogenic activity [22, 23]. Neovascularization and bone marrow microenvironment capacity to support the proliferation of tumor cells as well as the interactions between plasma cells and osteoclasts are important processes involved in MM pathogenesis [24]. VEGF is a potent mitogen for endothelial cells and is regarded as one of the most important molecules for its role in the vascularization of bone tissues [25]. Another factor with potent angiogenic activity is the HGF. HGF and its receptor c-met are expressed simultaneously in myeloma cells [26]. In MM patients elevated levels of HGF in the serum have been found to be associated with a poor prognosis [16, 27]. HGF appears to play a significant role in tumor angiogenesis by stimulating endothelial cell migration, proliferation, and capillary tube formation [28]. 

Three potential novel regulators of endothelial cell function and angiogenesis have been described. These are OPG and its ligands, RANKL and TNF-Related Apoptosis Inducing Ligand (TRAIL) [29]. In a recent study, it has been found that OPG is expressed in neovessels associated with malignant tumors and in angiogenic microvessels associated with inflammatory osteodestructive diseases [29–31]. Moreover, OPG with other angiogenic factors such as VEGF was found to have an additive effect on endothelial cell tube formation [32]. Since OPG is a decoy receptor for the above TNF family ligands, it seems that it exerts its angiogenic effect through inhibition of one or both of these molecules. In fact, using excess molar amounts of either TRAIL or RANKL, the angiogenic activity of OPG can be blocked. However, only RANKL was found to be an angiogenesis inhibitor by its own. So, RANKL reduces endothelial cell proliferation and induces their apoptosis, in vitro [13], whereas its role in regulating cell proliferation and survival differs between cell types, mainly due to the complex network of signals initiated by its interaction with RANK. IL-6 has been proposed as the major myeloma plasma cell growth factor. Furthermore, IL-6 has been shown to be a potent bone resorbing factor and may be involved in the production of bone lesions in MM patients [33]. 

In the present study, our results showed increasing serum levels of OPG in MM patients compared to the control group (*P* < 0.01). OPG serum levels show a tendency to decrease in advanced clinical stage and in patients with high score of bone disease (the lowest OPG serum levels were observed in patients with grade 3 bone disease). Higher levels of OPG were found in early clinical stages and in patients with minimal bone lesions (grade I). Probably, in patients without bone involvement, activity of ostepblasts is still coupled to the osteoclastic function. Osteoblast function decreases as the disease evolves and is the result of the suppressive effects of MM cells [34]. Our results are in agreement with others [11, 34] that OPG serum levels are elevated even in myeloma patients without bone lesions. In later stages of the disease, bone formation and osteoblast function are impaired which gives an explanation to the reduced OPG levels in patients with increased bone destruction [11, 34, 35]. No statistically significant difference was observed between OPG serum levels in pre and posttreatment MM patients and no significant correlation was observed with other parameters of disease activity at diagnosis and after treatment such as LDH, CRP, B2M, and IL-6. Recently, it has been reported that OPG blocks bone cancer-induced skeletal destruction after administration experimentally in mice with sarcoma-induced osteolysis [36]. Probably as reported recently the decreased level of OPG in myeloma bone disease gives a rationale for OPG as a new treatment for MM bone disease [37]. 

Furthermore, in our study we have shown that serum sRANKL levels were increased in MM patients compared to controls. This agrees with most other studies [34, 38]. RANKL and RANKL/OPG ratios, significantly increase with advancing disease stage and in patients with severe bone lesions. Furthermore, RANKL and RANKL/OPG ratios have positive correlations with clinical stage, grade of bone disease, angiogenic cytokines (HGF and VEGF), and factors of disease activity such as IL-6, B2M, and LDH. The only correlation of OPG serum levels was with RANKL/OPG ratio. 

To our best knowledge, no study until now has assessed a correlation between OPG, RANKL serum levels, their ratio RANKL/OPG and angiogenic cytokines such as HGF, VEGF, and IL-6 serum levels. In conclusion, OPG and RANKL are cytokines with a significant role in the pathophysiology of MM as regulators of bone turnover but also as significant mediators of angiogenesis. The exact role of OPG and RANKL in the mechanisms of angiogenesis and osteo-destructive disease in MM remains to be clarified.

## Figures and Tables

**Table 1:** Serum concentrations of measured parameters in healthy controls and patients with multiple myeloma.

	*n*	Mean ± SD	*P* (Mann Whitney)
*OPG* pmol/L			
Control	20	6.4 ± 5.3	< 0.03
Patients	54	12.5 ± 11.5	
*RANKL* pmol/L			
Control	20	13.54 ± 9.90	= 0.007
Patients	54	364.48 ± 393.63	
*RANKL/OPG*			
Control	20	14.44 ± 28.39	NS
Patients	54	51.69 ± 70.77	
*HGF* pg/mL			
Control	20	465.3 ± 163.4	< 0.001
Patients	54	1612.2 ± 1107.5	
*LDH* IU/L			
Control	20	163.1 ± 36.4	< 0.01
Patients	54	228.9 ± 77.2	
*VEGF* pg/mL			
Control	20	90.2 ± 14.4	< 0.001
Patients	54	272.3 ± 178.7	
*B2M* mg/dL			
Control	20	1.5 ± 0.6	< 0.001
Patients	54	3.9 ± 3.8	
*CRP* mg/L			
Control	20	0.4 ± 0.1	< 0.001
Patients	54	1.1 ± 1.3	
*IL-6* pg/mL			
Control	20	0.8 ± 0.5	< 0.001
Patients	54	6.4 ± 5.1	

**Table 2:** Mean ± SD values of the measured parameters in the group of MM patients in different disease stages.

	Stage	Mean ± SD	*P*
*OPG* (pmol/L)	1	16.5 ± 16.2	NS
	2	11.1 ± 9.2	
	3	10.4 ± 7.9	
*RANKL* pmol/L	1	72.01 ± 64.05	< 0.001
	2	235.29 ± 107.45	
	3	739.98 ± 445.84	
*RANKL/OPG*	1	7.62 ± 8.90	< 0.001
	2	48.63 ± 84.75	
	3	91.87 ± 63.71	
*HGF* (pg/mL)	1	804.3 ± 294.9	< 0.001
	2	1547 ± 469.1	
	3	2356.8 ± 1466.1	
*LDH* (IU/L)	1	180.4 ± 31.1	< 0.002
	2	218.5 ± 62.2	
	3	280.2 ± 88.9	
*VEGF* (pg/mL)	1	140.1 ± 105.0	< 0.001
	2	230.9 ± 141.8	
	3	425.1 ± 150.8	
*B2M* (mg/dL)	1	2.1 ± 0.9	< 0.001
	2	4.4 ± 5.3	
	3	4.8 ± 3.2	
*CRP* (mg/L)	1	1.5 ± 2.1	NS
	2	0.9 ± 0.7	
	3	1.1 ± 0.6	
*IL-6 *(pg/mL)	1	3.4 ± 2.1	< 0.001
	2	5.8 ± 2.7	
	3	9.5 ± 6.9	

**Table 3:** Mean ± SD values of the measured parameters in the group of MM patients according to the skeletal involvement.

	Bone involvement	Mean ± SD	*P*
*OPG* (pmol/L)	Low Score (Grade 0-1)	13.8 ± 13.6	NS
	High Score (Grade 2-3)	11.3 ± 9.3	
*RANKL* pmol/L	Low Score (Grade 0-1)	103.08 ± 79.29	< 0.001
	High Score (Grade 2-3)	589.84 ± 417.48	
*RANKL/OPG*	Low Score (Grade 0-1)	14.66 ± 21.97	< 0.001
	High Score (Grade 2-3)	83.61 ± 82.39	
*HGF* (pg/mL)	Low Score (Grade 0-1)	1150.4 ± 535.9	< 0.004
	High Score (Grade 2-3)	2010.2 ± 1312.0	
*LDH* (IU/L)	Low Score (Grade 0-1)	209.9 ± 74.7	0.05
	High Score (Grade 2-3)	245.3 ± 76.8	
*VEGF* (pg/mL)	Low Score (Grade 0-1)	170.7 ± 115.1	< 0.001
	High Score (Grade 2-3)	359.9 ± 178.7	
*B2M* (mg/dL)	Low Score (Grade 0-1)	2.9 ± 2.7	< 0.04
	High Score (Grade 2-3)	4.7 ± 4.5	
*CRP* (mg/L)	Low Score (Grade 0-1)	1.3 ± 1.7	NS
	High Score (Grade 2-3)	1.0 ± 0.7	
*IL-6* (pg/mL)	Low Score (Grade 0-1)	4.7 ± 4.9	< 0.001
	High Score (Grade 2-3)	7.8 ± 4.9	

**Table 4:** Correlation among RANKL and RANKL/OPG ratio with markers of angiogenesis and disease activity (*NS: not significant*).

		OPG	HGF	LDH	VEGF	B2M	CRP	IL-6
*RANKL*	*r*	NS	0.529	0.344	0.707	0.401	NS	0.422
	*P*	NS	< 0.0001	< 0.01	< 0.0001	< 0.003	NS	< 0.001
*RANKL/OPG*	*r*	−0.548	0.612	0.352	0.637	0.362	NS	0.390
	*P*	< 0.0001	< 0.0001	< 0.009	< 0.0001	< 0.007	NS	< 0.004

**Table 5:** Mean ± SD values of the measured parameters in MM patients before and after treatment.

		Mean ± SD	*P* (Wilcoxon test)
*OPG* (pmol/L)	Before treatment	11.5 ± 10.9	< 0.03
	After treatment	13.3 ± 14.0	
*RANKL * ** (**pmol/L)	Before treatment	335.10 ± 306.68	< 0.005
	After treatment	99.95 ± 108.52	
*RANKL/OPG*	Before treatment	76.55 ± 96.25	< 0.01
	After treatment	10.81 ± 9.91	
*HGF* (pg/mL)	Before treatment	1433.0 ± 1100.9	< 0.001
	After treatment	765.9 ± 210.5	
*LDH* (IU/L)	Before treatment	218.6 ± 76.0	< 0.002
	After treatment	176.1 ± 33.4	
*VEGF* (pg/mL)	Before treatment	243.8 ± 177.0	< 0.001
	After treatment	120.7 ± 40.4	
*IL-6* (pg/mL)	Before treatment	3.5 ± 3.6	< 0.003
	After treatment	2.4 ± 1.2	
